# Malignant peripheral nerve sheath tumor in the pelvis: a case report

**DOI:** 10.1186/s40792-023-01733-5

**Published:** 2023-09-06

**Authors:** Rika Ono, Tetsuro Tominaga, Takashi Nonaka, Yuma Takamura, Kaido Oishi, Toshio Shiraishi, Shintaro Hashimoto, Keisuke Noda, Terumitsu Sawai, Takeshi Nagayasu

**Affiliations:** Department of Surgical Oncology, Nagasaki University Graduate School of Biomedical Science, 1-7-1 Sakamoto, Nagasaki, 852-8501 Japan

**Keywords:** Malignant peripheral nerve sheath tumors, Neurofibromatosis type 1, Surgery

## Abstract

**Background:**

Malignant peripheral nerve sheath tumors (MPNSTs) are malignancies that arise or differentiate from or infiltrate peripheral nerves and account for approximately 5% of soft-tissue malignancies. Approximately half of MPNSTs develop in patients with neurofibromatosis type 1 (NF1), a hereditary disease. MPNSTs occur mainly in the trunk, proximal extremities, and neck, but can on rare occasion arise in or near the gastrointestinal tract, and intestinal complications have been reported. We describe herein a case with resection of an MPNST arising in the pelvic region.

**Case presentation:**

A 51-year-old woman had undergone repeated resections for systemic neurofibrosis associated with NF1. This time, a pelvic tumor was noted on follow-up positron emission tomography computed tomography (CT). She presented with slowly progressive radiating pain in the lower extremities and was referred to our hospital for tumor resection. Contrast-enhanced CT showed a 75 × 58-mm mass in the right greater sciatic foramen directly below a 24 × 28-mm mass. Open pelvic tumor resection was performed for pelvic neurofibroma. The obturator nerve was identified lateral to the main tumor and the sciatic nerve was identified dorsally, then dissection was performed. The closed nerve was spared, while the sciatic nerve was partially dissected and the two tumors were removed. Both tumors were elastic and hard. Pathologic findings were MPNST for the large specimen and neurofibroma with atypia for the small specimen. The patient developed temporary postoperative ileus, but is generally doing well and is currently free of recurrence or radiating pain. The patient is at high risk of recurrence and close monitoring should be continued.

**Conclusions:**

We encountered a rare case of MPNST. Due to the high risk of recurrence, surgery with adequate margins was performed, with a requirement for appropriate follow-up.

## Background

Malignant peripheral nerve sheath tumors (MPNSTs) account for approximately 5% of all soft-tissue sarcomas, and about half occur in patients with neurofibromatosis-1 (NF1) [1]. NF-1 occurs in 1 in 3000–5000 people, among whom MPNSTs are relatively rare, at 2–5% [2, 3]. In addition, MPNSTs predominantly arise in the proximal extremities, trunk, head and neck, and rarely in the pelvis [3]. We describe herein the resection of an intrapelvic MPNST in a patient with NF1.

## Case presentation

**Fig. 1:**
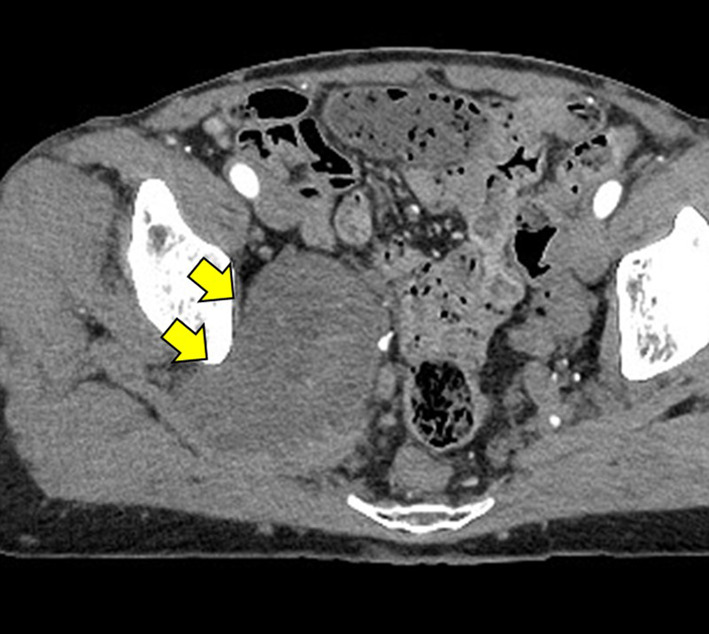
Pelvic CT. Pelvic CT shows a heterogeneous, internal, 75-mm-diameter mass in the right greater sciatic foramen

**Fig. 2:**
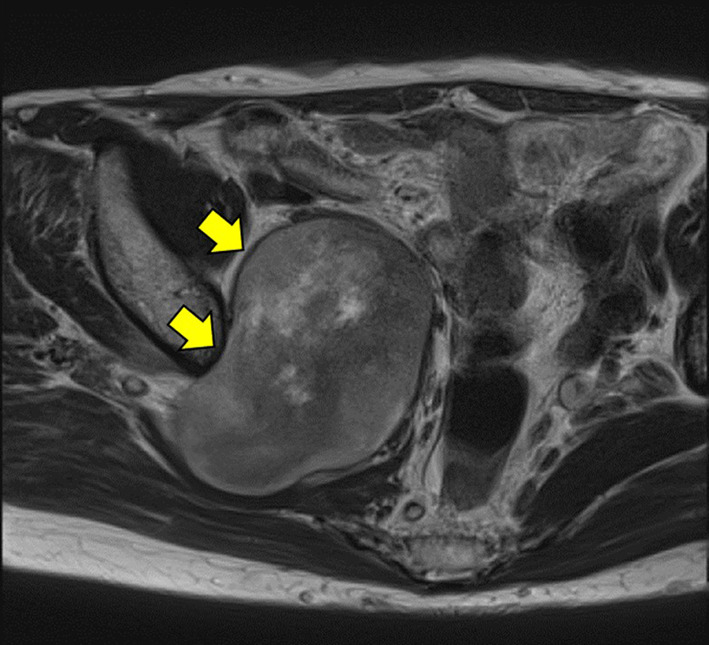
Pelvic MRI. Pelvic MRI shows a heterogeneous high-signal mass on T2-weighted imaging

**Fig. 3:**
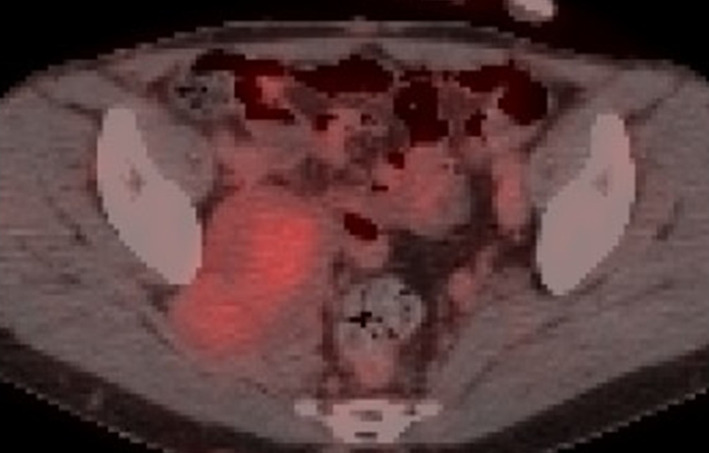
PET–CT. PET–CT shows high uptake (SUVmax 12.7) consistent with a pelvic mass

**Fig. 4:**
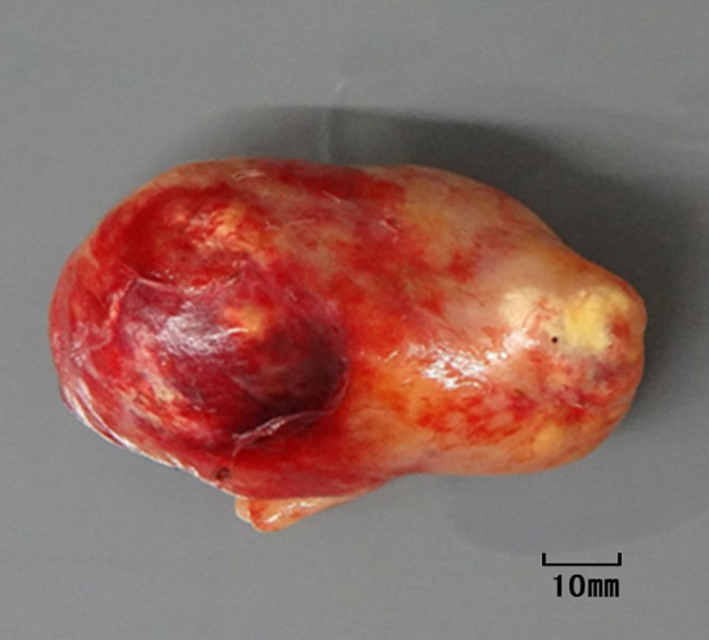
The excised specimen. The specimen is a yellowish-white mass with elastic hardness, 80 mm in diameter and with a solid part on the cut surface

**Fig. 5:**
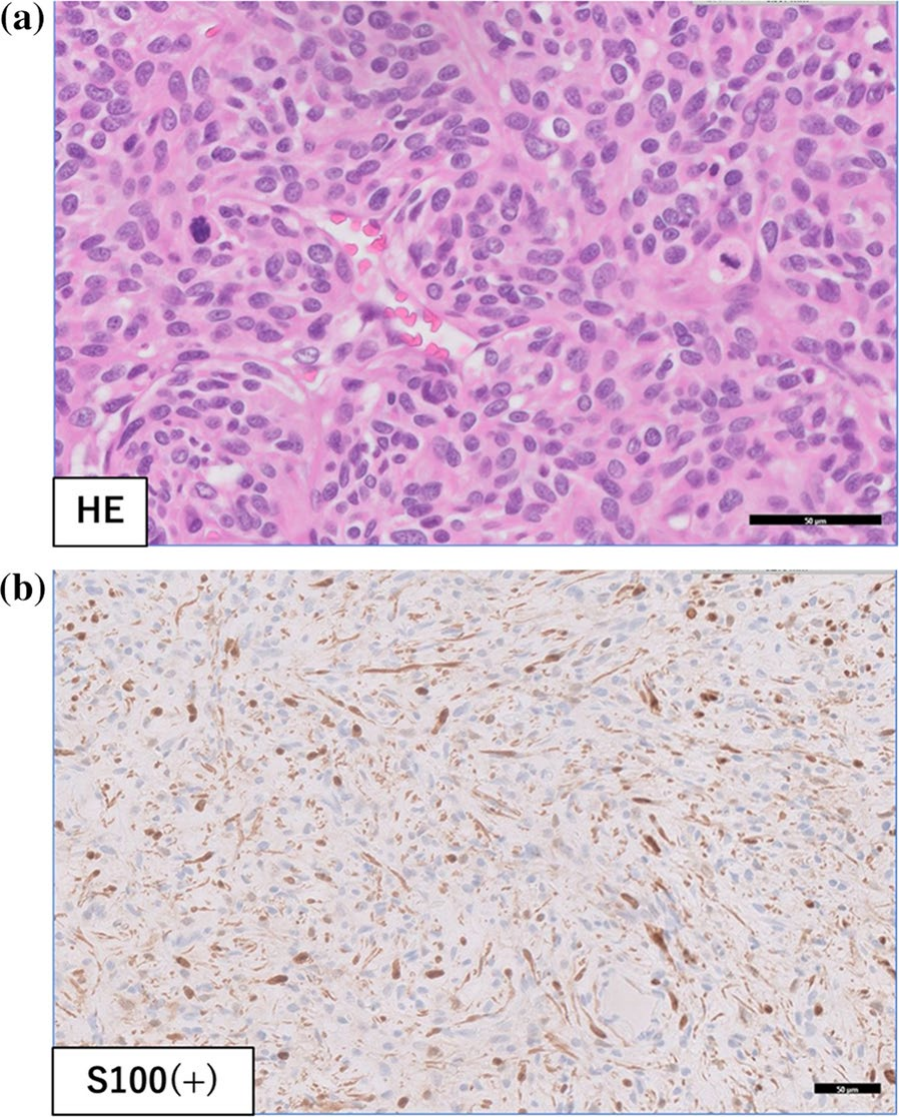
**a** Histopathological examination. A coarse, dense pattern of cells with bundles of spindle-shaped cells with atypia and hyperplastic nuclei with darkly stained pleomorphic swelling. **b** Immunostaining. The atypical cells are S100-positive, and malignant peripheral nerve sheath tumor is diagnosed

The patient was a 51-year-old woman on previous medical follow-up with NF1. A pelvic mass was noted on follow-up PET–CT. She was referred to our hospital for close examination and treatment due to slowly progressive radiating pain in the right lower extremity. The patient had no contributory family history, but had undergone several previous tumor resections associated with NF1. Contrast-enhanced CT showed a heterogeneous mass 75 mm in diameter in the right greater sciatic foramen (Fig. 1). Pelvic MRI showed a heterogeneous, signal-hyperintense mass on T2-weighted imaging (Fig. 2), and PET–CT showed an area of high uptake (SUVmax 12.7) consistent with the pelvic mass identified on MRI (Fig. 3). Surgery was performed under a presumptive diagnosis of slowly increasing intrapelvic neurofibroma. The surgical approach was determined in consultation with an orthopedic surgeon before surgery. The plan was to use an open approach with the option of adding a posterior approach depending on tumor mobility and the state of the visual field near the sciatic foramen. Open surgery was initiated through a midline incision in the lower abdomen. The tumor located in the right lateral region was elastic and firm. The internal iliac vein was exposed and seen to be pushed up by the tumor, and was partially ligated and dissected. The obturator nerve and sciatic nerve were identified dorsally. A partial connection was apparent between the caudal side of the tumor and the sciatic nerve. A portion of the sciatic nerve was resected in combination with the tumor, preserving the sciatic nerve trunk. The obturator nerve was preserved with no connection to the tumor. The excised specimen was a yellowish-white mass with elastic hardness, 80 mm in diameter and with a solid part on the cut surface (Fig. 4). Histological examination showed a coarse, dense pattern of cells with bundles of spindle-shaped cells with atypia and hyperplastic nuclei with darkly stained pleomorphic swelling (Fig. 5a). No clear necrotic foci were evident in the tumor, but scattered fission images were seen in areas of high cell density. Immunostaining showed that the atypical cells were S100-positive, so MPNST was diagnosed (Fig. 5b). Postoperatively, the patient developed mild and temporary bowel obstruction, which resolved with conservative treatment. As of the time of writing, 8 months postoperatively, no evidence of recurrence has been seen.

## Discussion

MPNSTs are soft-tissue malignancies that arise or differentiate from or infiltrate peripheral nerves and account for approximately 6% of all soft-tissue sarcomas [1]. The peak age of onset is between 30 and 40 years, and around half of cases arise in patients with NF1 [4]. NF1 is characterized by multiple neurofibromas in the skin and nerves, and malignant transformation of these neurofibromas is believed to be the mechanism of tumorigenesis for MPNST arising against the background of NF1 [4].

Kim et al. reviewed 389 cases of peripheral schwannoma [5]. Of these, 28 (7.2%) were MPNSTs, but none occurred in the pelvis. Kar et al. also reported 24 cases of MPNST, with 2 (8.3%) showing a pelvic origin [6].

About 70% of cases were detected as painless swellings, and clinical symptoms are said to become apparent as the tumor grows [7]. In particular, pelvic tumors often grow asymptomatically, and this case showed no significant symptoms other than slow progression of radiating pain in the lower extremities.

Yun et al. reported on the usefulness of MRI for diagnosing MPNST [8, 9]. They reported a minimum ADC of 0.73 ± 0.47 × 10^–3^ mm^2^/s for benign nerve sheath tumors and 1.12 ± 0.37 × 10^–3^ mm^2^/s for MPNST. Bredella et al. also reported on the utility of PET–CT [10]. They reported that SUVmax for benign schwannoma ranged from 0 to 5.3 (mean, 1.5 ± 0.37), while that for malignant cases ranged from 3.8 to 13.0 (mean, 8.5 ± 0.63) [10, 11]. In the present case, preoperative MRI showed a high ADC of 1.54 × 10^–3^ mm^2^/s, and PET–CT showed an SUV of 12.7, suggestive of malignancy when examined retrospectively.

Definitive diagnosis was reached on histopathological examination, which showed dense bundles of atypical spindle-shaped cells [12]. The chromatin-rich, oblong nuclei showed multiple mitotic structures, often with internal necrosis and hemorrhage. Immunostaining showed positive results for S-100 protein in more than half of the cases [13]. Histologically, the present case comprised spindle-shaped bundles of cells with a vascular pattern similar to hemangiopericytoma and a coarse, dense pattern of cells. The histology was diverse, including map-like necrosis and a prominent nuclear fission picture. Immunostaining was also positive for S-100 protein, and MPNST was therefore diagnosed.

Various treatment options are available for MPNST, including doxorubicin plus ifosfamide and peripheral blood stem cell transplantation. However, no clear benefits from these have been reported, and resistance to radiation has also been described [12, 14, 15]. The first-choice treatment is therefore complete surgical resection. However, depending on the site, adequate margins can be difficult to achieve, and 40–65% of cases recur locally [4, 12, 16]. As a result, the 5-year survival rate for NF1-associated MPNST, as in the present case, is poor, at 16–30% [3, 4, 17], with a median time to recurrence of 6–9 months and a median time to distant metastasis of 8–9 months [13, 18]. Prognostic factors include tumor diameter ≥ 5 cm, positive dissection margins [19], along with tumor size for tumors outside the extremities, such as in the trunk, head, or neck [20]. In the present case, the tumor was a 75-mm-diameter MPNST arising in the pelvis in association with NF1, and the prognosis was considered poor. The dissection margins were negative and no evidence of recurrence has been seen as of 8 months postoperatively. Although no clear consensus has yet been reached regarding follow-up, this patient is considered to be at high risk of recurrence and will be followed up for recurrence with CT every 3 months.

## Conclusions

We encountered a rare case of MPNST. Due to the high risk of recurrence, surgery with adequate margins was performed, with a requirement for appropriate follow-up.

## Abbreviations

MPNST: Malignant peripheral nerve sheath tumor
NF1: Neurofibromatosis type 1

## Declarations

### Availability of data and materials

The data that support the findings of this study are available from the corresponding author upon reasonable request.
